# Evolution of tumor cells during AsiDNA treatment results in energy exhaustion, decrease in responsiveness to signal, and higher sensitivity to the drug

**DOI:** 10.1111/eva.12949

**Published:** 2020-04-11

**Authors:** Pierre‐Marie Girard, Nathalie Berthault, Maria Kozlac, Sofia Ferreira, Wael Jdey, Srividya Bhaskara, Sergey Alekseev, Frederic Thomas, Marie Dutreix

**Affiliations:** ^1^ Institut Curie CNRS INSERM UMR 3347 PSL Research University Orsay France; ^2^ CNRS INSERM UMR 3347 Université Paris‐Sud Université Paris‐Saclay FOrsay France; ^3^ Onxeo Paris France; ^4^ Huntsman Cancer Institute University of Utah School of Medicine Salt Lake City Utah USA; ^5^ CREEC/MIVEGEC UMR IRD 224‐CNRS 5290 Université de Montpellier Montpellier France

**Keywords:** biomedicine, disease biology, evolutionary theory, transcriptomics

## Abstract

It is increasingly suggested that ecological and evolutionary sciences could inspire novel therapies against cancer but medical evidence of this remains scarce at the moment. The Achilles heel of conventional and targeted anticancer treatments is intrinsic or acquired resistance following Darwinian selection; that is, treatment toxicity places the surviving cells under intense evolutionary selective pressure to develop resistance. Here, we review a set of data that demonstrate that Darwinian principles derived from the “smoke detector” principle can instead drive the evolution of malignant cells toward a different trajectory. Specifically, long‐term exposure of cancer cells to a strong alarm signal, generated by the DNA repair inhibitor AsiDNA, induces a stable new state characterized by a down‐regulation of the targeted pathways and does not generate resistant clones. This property is due to the original mechanism of action of AsiDNA, which acts by overactivating a “false” signaling of DNA damage through DNA‐PK and PARP enzymes, and is not observed with classical DNA repair inhibitors such as the PARP inhibitors. Long‐term treatment with AsiDNA induces a new “alarm down” state in the tumor cells with decrease in NAD level and reactiveness to it. These results suggest that agonist drugs such as AsiDNA could promote a state‐dependent tumor cell evolution by lowering their ability to respond to high “danger” signal. This analysis provides a compelling argument that evolutionary ecology could help drug design development in overcoming fundamental limitation of novel therapies against cancer due to the modification of the targeted tumor cell population during treatment.

## 1 INTRODUCTION

It is increasingly acknowledged that cancer is an ecological and evolutionary process (Merlo, Pepper, Reid, & Maley, 2006; Ujvari, Roche, & Thomas, 2017), and that cancer therapies must therefore become as adaptive and dynamic as the system they are fighting. This requires application of Darwinian principles not only to understand the processes that lead to phenotypic adaptation of malignant cells becoming insensitive to therapies, but also to anticipate the evolutionary responses of cancers to treatments in a way that permits “evolutionarily enlightened” cancer therapies (Cunningham, Gatenby, & Brown, 2011). Although promising, speculation has however until now proven more attractive than empirical evidence, and to our knowledge, only adaptive therapy (Gatenby, Brown, & Vincent, 2009; Zhang, Cunningham, Brown, & Gatenby, 2017) focuses on exploiting ecological (changes in the tumor size) and evolutionary dynamics (changes in the frequency of different cancer cell phenotypes) to delay or prevent the proliferation of resistant phenotypes (Bacevic et al., 2017). Here, we propose another therapeutic perspective directly inspired from ecological and evolutionary principles, namely a strategy exploiting some properties of the “smoke detector principle” developed in evolutionary medicine and behavioral ecology (Nesse, 2005).

Conventional anticancer treatments and, more recently, targeted therapies have improved the control of tumors. Nonetheless, the rate of therapy failure is high, primarily due to side effects that limit dose escalation and to the onset of resistance during treatment. Targeting tumors based on genetic defects has revolutionized the era of cancer treatment and precision medicine. Targeted therapies yield high rates of initial response, although most responding tumors fail to achieve a complete response. Furthermore, the development of acquired resistance is nearly universal in patients who respond initially to therapy. For example, chemotherapeutics often produce remission for only a limited period, because intrinsic or acquired resistance to treatment by the malignant cells leads to relapse, tumor progression, and death (Gatenby & Brown, 2018; Holohan, Van Schaeybroeck, Longley, & Johnston, 2013; Michor, Nowak, & Iwasa, 2006). Although the ability of cancer to evolve has been traditionally perceived as a major problem in curing it, it has been increasingly suggested that it could also inspire novel therapies.

The use of “fake drugs” was first proposed by the group of Gatenby to activate the efflux pumps in resistant cells and cause them to expend energy without actually giving them a survival benefit over nonresistant cells (Kam et al., 2015). Another kind of “fake drug,” based on the DNA bait (DBait) concept, has been recently proposed which uses agonists of enzymes that signal DNA damage in the cell in order to inhibit DNA repair (Croset et al., 2013; Jdey, Thierry, Popova, Stern, & Dutreix, 2017; Quanz, Chassoux, et al., 2009; Quanz et al., 2012). AsiDNA molecules are small double‐stranded DNA molecules that mimic double‐strand breaks. They bait DNA damage signaling enzymes and trigger a massive false signal, preventing DNA repair enzyme activity. Defects in DNA repair are associated with genetic instability and thus may promote mutagenesis and facilitate the emergence of resistance. Indeed, poly(ADP‐ribose) polymerase (PARP) inhibitors, which have been tested for many years and have become a potential supplement to conventional chemotherapy, show increasing evidence of the appearance of resistance during treatment (Kim et al., 2017). In a recent work, it was reported that continuous or cyclic treatments with AsiDNA are much less prone to promote resistance emergence than regular DNA repair inhibitors (Herath et al., 2019; Jdey et al., 2019). In contrast, treated populations showed increased sensitivity to AsiDNA. In this review, we summarize many published and new data that describe the new state of the treated populations and propose that they could have evolved to decrease their response to the agonist activity of AsiDNA on its PARP and DNA‐PK targets. As proposed in the theory of the “smoke detector principle,” the cell populations evolved to decrease the highly energy consuming activation of DNA‐PK and PARP at the expense of being less able to face changes in environment or new stresses.

Here, we use the results obtained with the MDA‐MB‐231 cell line to illustrate our demonstration and propose a new mode of tumor evolution that we consider driven by the equilibrium between risk and energy cost. Many of the results like acquisition of sensitivity to AsiDNA after long‐term treatment, change in chromatin, transcriptome modifications, and depletion in nicotinamide adenine dinucleotide (NAD) have been reproduced in several other cell lines.

## 2 MATERIALS AND METHODS

All chemicals were provided by Sigma‐Aldrich.

### 2.1 AsiDNA molecules

AsiDNA is a 64‐nucleotide (nt) oligodeoxyribonucleotide consisting of two 32‐nt strands of complementary sequence connected through a 1,19‐*bis* (phospho)‐8‐hydraza‐2‐hydroxy‐4‐oxa‐9‐oxo‐nonadecane linker, with a cholesterol at the 5′‐end and three phosphorothioate internucleotide linkages at each of the 5′ and the 3′ ends (Agilent). The sequence is as follows: 5′‐ X GsCsTs GTG CCC ACA ACC CAG CAA ACA AGC CTA GA L ‐ C_L_TCT AGG CTT GTT TGC TGG GTT GTG GGC AC sAsGsC ‐3′, where **L** is an amino linker, **X** a cholesteryl tetraethyleneglycol, **C_L_** a carboxylic (hydroxyundecanoic) acid linker, and **s** a phosphorothioate linkage). Cy5‐tagged AsiDNA is derived from AsiDNA by coupling Cy5 fluorescent tag to **C_L_**.

### 2.2 Cell lines, treatment, and survival measurement

Triple‐negative human breast cancer cells (MDA‐MB‐231, ATCC^®^ HTB‐26™) and normal human breast cells (MCF 10A, ATCC^®^ CRL‐10317™) were purchased from ATCC. MDA‐MB‐231 were grown in L‐15 medium supplemented with 10% fetal calf serum (FCS) and 100 U/ml penicillin/100 μg/ml streptomycin (P/S), and maintained at 37°C in a humidified atmosphere at 0% CO_2_. MCF 10A were grown in MEBM^TM^ Basal Medium (Lonza, CC‐3151) supplemented with 1× MEGM^TM^ SingleQuotsTM Supplement Pack (Lonza, CC‐4136). Treatment protocol to generate “evolved” population was 6 weeks alterning three times, 1 week with AsiDNA and 1 week recovery. In brief, cells were seeded in six‐well culture plates with 2.10^4^ cells per well and incubated 24 hr at 37°C before addition of the drug (5 µM AsiDNA). Cells were harvested on day 6 after treatment, washed, and counted after staining with 0.4% trypan blue (Sigma‐Aldrich). After counting, cells were seeded in six‐well culture plates, medium was changed 24 hr after incubation to remove dead cells, and the cells were allowed to recover for six more days. Another cycle of treatment/recovery was then started for up to three cycles. The so called “naïve” populations were grown in parallel in similar conditions without addition of AsiDNA. Survival was estimated at the end of the week of growth with AsiDNA by counting living cells with trypan blue. The survival is the ratio of the number of cells grown with AsiDNA on the number of cells grown without AsiDNA.

#### 2.2.1 Quantification of γH2AX by Western blot

In brief, cells were boiled in sodium dodecyl sulfate sample buffer (50 mM Tris–HCl, pH 6.8, 1% b‐mercaptoethanol, 2% sodium dodecyl sulfate, 0.1% bromophenol blue and 10% glycerol). Proteins were separated by electrophoresis in 12% acrylamide/bisacrylamide (37.5/1) gels, transferred to nitrocellulose membranes, blocked with Odyssey buffer for 1 hr, and hybridized overnight at 4°C with primary mouse monoclonal anti‐γH2AX antibody (clone JBW301, Merck Millipore) or anti‐γ‐actin antibody (clone AC‐15, Sigma). Blots were then incubated with secondary goat anti‐mouse IR Dye 800CW antibody (LI‐COR), and protein–antibody complexes were revealed on Odyssey (LI‐COR Biotechnology). Quantifications were performed using Odyssey software.

#### 2.2.2 Quantification of γH2AX by Flow cytometry

In brief, cells were fixed in 4% paraformaldehyde (PAF), permeabilized in 0.25% Triton X‐100 in phosphate‐buffered saline (PBS) for 20 min on ice, washed with PBS, incubated 20 min with blocking solution (PBS containing 10% FCS, 0.3 M glycine and 0.05% sodium azide) for 30 min at room temperature, and washed in PBS containing 1% FCS, 1 mM EDTA and 0.09% sodium azide (AutoMACS Running Buffer, Miltenyi Biotech) before to be incubated in MACS buffer containing Alexa Fluor 647 Mouse anti H2AXpS139 (BD Pharmingen, clone N1‐431) diluted at 1/50 or isotype APC mouse IgG1 (Miltenyi Biotech, ref. 130‐113‐196) diluted at 1/50. Incubation was performed at room temperature in the dark for 2 hr with gentle mixing every 30 min. Fluorescence intensity of each sample was recorded on a FACS LSRFortessa™ X‐20 (BD Biosciences), and the data were analyzed using FlowJo software (Tree Star).

#### 2.2.3 Quantification of AsiDNA cellular uptake

To assess for AsiDNA uptake by the cells, Cy5‐tagged AsiDNA was incubated with the cells and fluorescence was recorded by flow cytometry 24 hr postincubation.

#### 2.2.4 NAD content

NAD content was determined using the NAD/NADH‐Glo Assay kit (Promega G9071) according to the manufacturer's instructions. The resulting luminescent signals were measured on a microplate reader (Victor™ X3, Perkin Elmer).

#### 2.2.5 Proliferation

Cell proliferation assays were done over a period of 15 days. Approximately 20.000 cells per well were plated in a 12‐well plate per cell line. Viable cell counts were performed by trypan blue exclusion on days 2, 5, 6, 9, 12, and 15.

#### 2.2.6 Cell cycle analysis

Cell cycle progression was assessed using the BrdU pulse method. MDA‐MB‐231 cells were pulse‐labeled with BrdU for 1 hr and allowed to recover for different incubation periods (0, 4, 8, 24, or 48 hr). Cells were fixed in 70% cold ethanol overnight at −20°C, permeabilized on ice in PBS‐0.5% Triton X‐100 for 15 min, washed in PBS, and incubated with anti‐FITC‐BrdU antibody in 2% BSA in PBS. After washing with PBS, cells were incubated with an Alexa Fluor 488‐conjugated secondary antibody, washed and resuspended in 0.5 ml of PBS containing 1% FBS, 1 mg/ml RNaseA, and 50 μg/ml propidium iodide. FACS analyses were performed on a BD FACSCANTO II flow cytometer. Data were analyzed using FlowJo software (Tree Star).

### 2.3 PAR quantification by ELISA assay

Cells were lysed using a cell scraper and boiled for 10 min in PathScan Sandwich ELISA Lysis Buffer (Cell Signaling Technology, 7018) and 1 mM PMSF (phenylmethanesulfonyl fluoride; Sigma, P7626) after treatment. Quantification of total protein was performed using Pierce BCA assay (Pierce, 23250). Wells of opaque 96‐well plates (Pierce, 15042) were coated (overnight, 4°C) with 4 μg/ml mouse anti‐PAR (Trevigen, 4335) in carbonate buffer (0.015 M Na_2_CO_3_, 0.035 M NaHCO_3_, pH 9.6), washed 6 times with PBS 1× + 0.1% Tween (PBST), and were then blocked with Superblock buffer (Thermo Scientific, W6423W) for 1 hr at 37°C. Samples (three dilutions to 0.05–0.4 mg/ml of proteins in Superblock buffer) were loaded and incubated overnight at 4°C. After washing 8 times with PBST, 75 μl of rabbit anti‐PAR (Trevigen, 4336), diluted 1/1000 in PBS 1× + 2% milk + 1% mouse serum, was added for 1 hr at room temperature. After washing 8 times with PBST, 75 µl of anti‐rabbit HRP‐conjugated antibody (Abcam, ab97085), diluted 1/5000 in PBS 1× + 2% milk + 1% mouse serum, was added (1 hr, room temperature), and after washing 8 times with PBST, the reaction was visualized by the addition of 75 μl per well of Supersignal Pico (Pierce, 37070). The reaction was read using Victor plate reader at 425 nm 10 min after addition. As a reference for quantification, a standard curve was established by a 1:2 serial dilution of pure PAR (Enzo, ALX‐202‐043) in Superblock buffer to final concentrations of 7.8–32,000 pg/ml.

### 2.4 Statistical analysis

All statistical analyses were performed using the two‐tailed Student *t* test.

## 3 RESULTS

### 3.1 Molecular and cellular consequences of AsiDNA treatment

**Figure 1:**
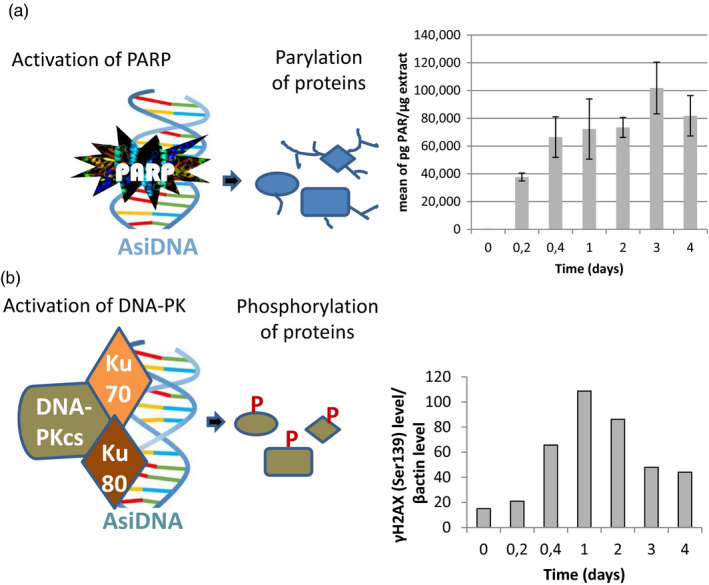
Kinetics of enzyme activation by AsiDNA. Cells were treated for different times with AsiDNA. (a) PARP activation revealed by poly‐adenyl‐ribose quantification in cell extracts. (b) H2AX phosphorylation quantified by Western blot

AsiDNA belongs to a unique class of DNA repair inhibitors that act by overactivating PARP and DNA‐PK in tumor cells. The activity of these enzymes is revealed by the modification of many targets in the cytoplasm and in the nucleus. Phosphorylation of the histone H2AX (Quanz, Berthault, et al., 2009) and the chaperone protein HSP90 (Quanz et al., 2012) are the most abundant targets of the DNA‐dependent kinase DNA‐PK shown to be activated by AsiDNA. Here, we showed that AsiDNA triggers phosphorylation of H2AX in MDA‐MB‐231 cells (Figure 1b). In parallel, accumulation of poly(ADP‐ribose) polymers produced by the activated PARP enzyme is monitored after AsiDNA treatment (Figure 1a). For both enzymes, the activity induced by AsiDNA persists throughout the time of exposure to AsiDNA.

**Figure 2:**
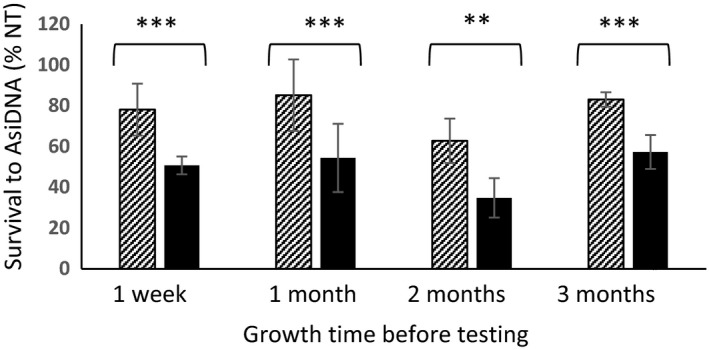
Survival to repeated treatments: MDA‐MB‐231 «evolved» (black) and «naïve» (dashed) populations were frozen after the end of treatment. They were unfrozen and grown for the indicated periods of time before monitoring survival to 1 week of AsiDNA treatment. (a) schema of treatment (number of independent populations = 5–8). *p* values (***, *p* < .007; **, *p* < .01)

As it was already described, cyclic treatments with AsiDNA induce a progressive increase in the sensitivity of cells to AsiDNA (Jdey et al., 2019). Interestingly, the difference in sensitivity between cells having survived to three cycles of treatment (“evolved”) and their counterpart grown in similar conditions without AsiDNA treatment (“naïve”) lasts for several weeks after the end of treatment indicating that such sensitization is a stable condition (Figure 2a).

### 3.2 Characterization of the “evolved” cells

**Figure 3:**
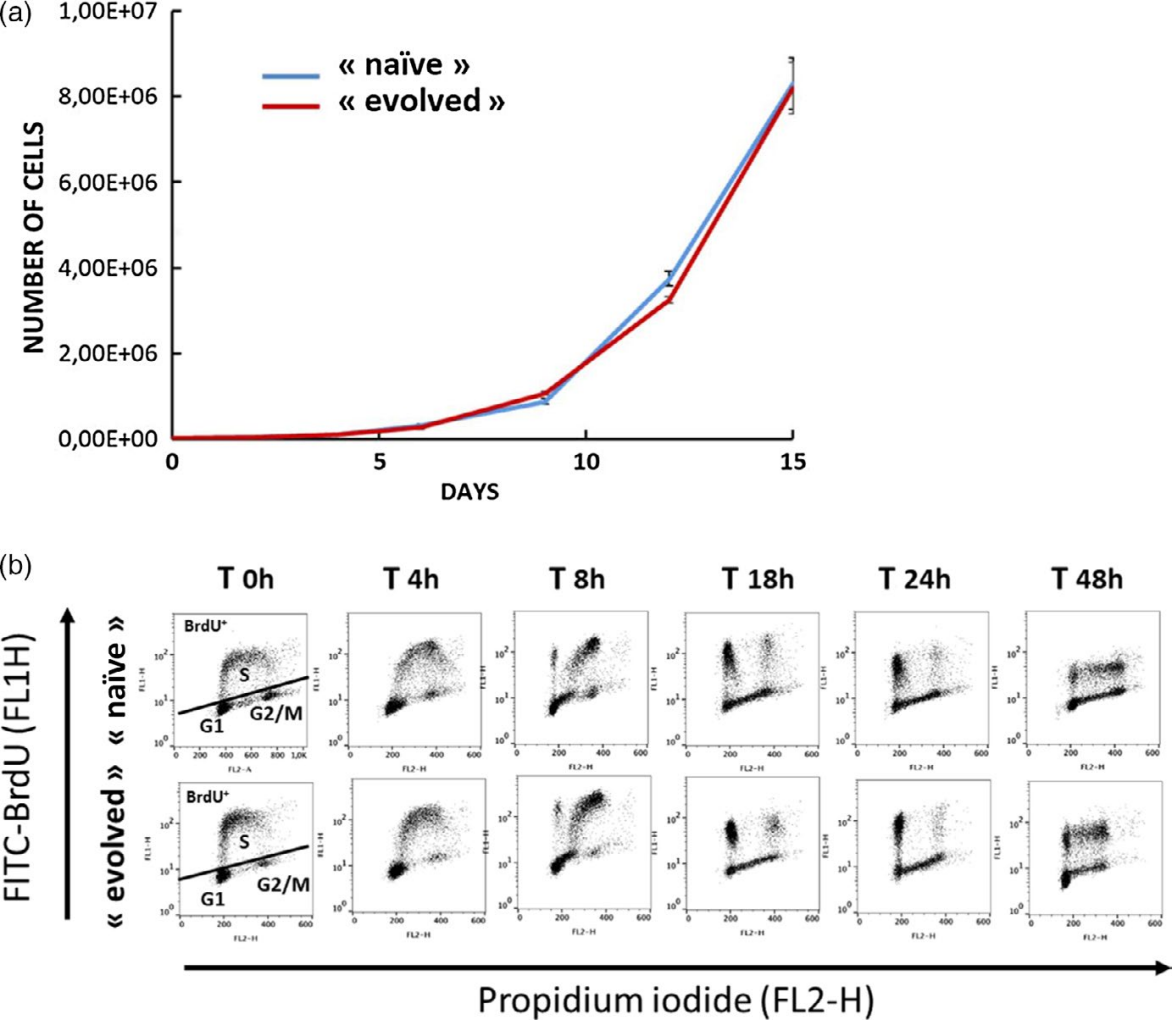
Cell growth and cell cycle distribution of «naïve» and «evolved» cells. (a) «Naïve» (blue line) and «evolved» (red line) cells were allowed to grow for up to 2 weeks and counting every 2 days. Shown is the average ± *SD* of 14 independent cultures. (b) «Naïve» (upper panels) and «evolved» (lower panels) were pulse‐labeled with BrdU and then released in BrdU‐free medium. Cells were fixed at various time points from 0 to 48 hr postlabeling and analyzed by flow cytometry. BrdU‐positive cells (BrdU^+^) are cells that were in S‐phase at the time of labeling (T0 hr). Propidium iodide labeling monitors the DNA content of the cells at the time of analysis (indicated above the panels) that double between G1 and G2/M

**Figure 4:**
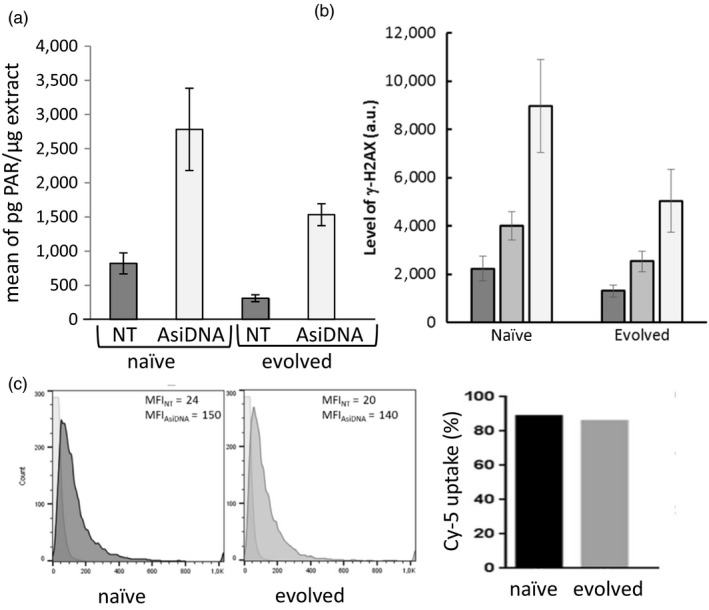
Activation of PARP and DNA‐PK by AsiDNA. (a) PAR quantification and (b) γ‐H2AX phosphorylation in cells treated with 5 µM (light gray) or 10 µM (white) AsiDNA or untreated (dark gray). Shown is the average ± *SD* of 3 independent experiments. (c) Uptake of Cy5‐tagged AsiDNA in “naïve” and “evolved” cells. Left panels: FACS analysis; right panel: mean value of fluorescence quantification

To identify if the “evolved” cell populations could result from a clonal selection of cells better fitted to the growth conditions though more sensitive to the drug, we compared the growth and the cell cycle of “evolved” and “naïve” populations. Observation of 14 independent cell cultures during 2 weeks did not reveal any difference in growth between “naïve” and “evolved” populations (Figure 3a). In agreement with this result, no significant differences were observed between the populations when monitoring cell cycle progression (Figure 3b). We compared genomes of the two groups and did not identify clear modifications that would suggest a clonal evolution (Table S1). We analyzed the ability of AsiDNA to activate PARP or DNA‐PK in “naïve” and “evolved” populations. DNA‐PK activation was estimated by monitoring phosphorylation of the histone variant H2AX (Figure 4b), and PARP activation was estimated by the poly(ADP‐ribose) (PAR) content of cells (Figure 4a). “Evolved” populations showed a lower activation of both enzymes at basal level as well as after AsiDNA treatment as compared to “naïve” populations. Using fluorescent cy5‐AsiDNA, we verified that AsiDNA uptake was similar in both cell lines and could not explain the low activation observed in “naïve cells” (Figure 4c). Therefore, the reduced activity in “evolved” cells suggests a decrease in responsiveness to AsiDNA in cells previously exposed to repetitive treatments with these molecules.

### 3.3 Altered micrococcal nuclease sensitivity in “evolved” cells

**Figure 5:**
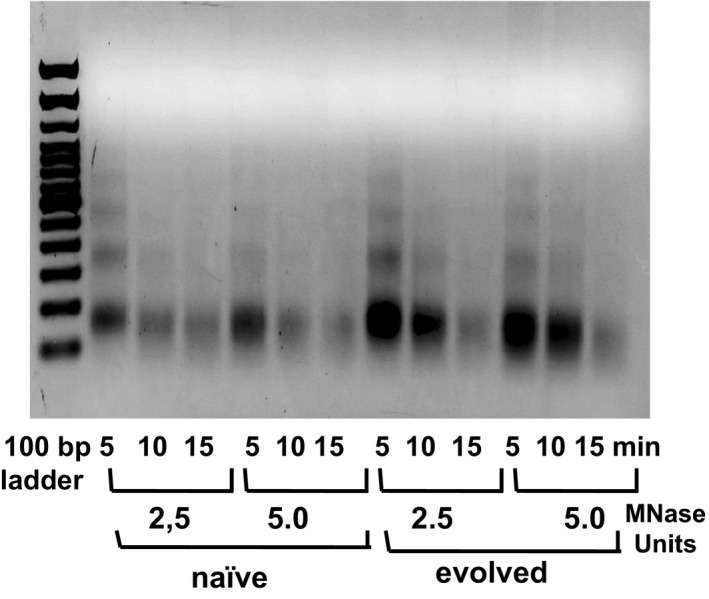
Chromatin sensitivity to Mnase. Nuclei (10^5^) isolated from “naïve” and “evolved” populations were digested with 2.5 and 5 units Mnase for 5, 10, and 15 min, and purified DNA was electrophoresed on a 1.5% agarose gel. According to Kozlak et al. (manuscript in preparation)

The fact that all independent populations seem to evolve similarly without indication of a specific clonal expansion has led us to further investigate the possibility of an epigenetic change induced by the treatment. We analyzed the global chromatin by monitoring its sensitivity to digestion by a micrococcal nuclease. The difference in chromatin structure between “naïve” and “evolved” cell chromatin was revealed by a lower sensitivity to nuclease digest of the “evolved” mononucleosomes (Figure 5). Indeed, the “evolved” chromatin showed reproductively a lower sensitivity to micrococcal nuclease than “naïve” chromatin with a higher persistence of the monomers after prolonged digestion (Figure 5), suggesting a possible alteration in the chromatin state in evolved cells.

### 3.4 Metabolism decrease in “evolved” cells

**Figure 6:**
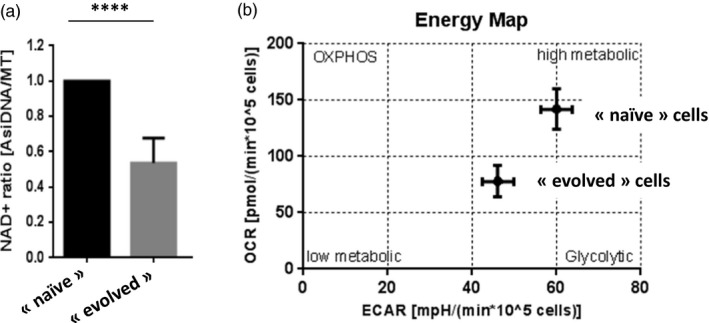
Reduced metabolic activity in “evolved” cells. (a) Reduced basal level of NAD in “evolved” cells compared to “naïve” cells. (b) Ratio of OCR:ECAR quadrant showing the bioenergetics phenotype of “naïve” and “evolved” cell lines. All Seahorse experiments (Seahorse Agilent) were performed for a minimum of 3 times in triplicates. According to Kozlak et al. (manuscript in preparation)

Chromatin structure change is often associated with large transcriptional modifications. Actually, transcriptome analysis revealed a major change in gene expression with a large excess of genes down‐regulated in 3 independent “evolved” populations as compared to three independent “naïve” populations (Table S2; access to raw data in GEO; GSE144023). A similar bias to repressed expression was observed in MDA‐MB‐231 tumors xenografts after three cycles of AsiDNA injection (Jdey et al., 2019). Strikingly, metabolic pathways were significantly deregulated in “evolved” cells as compared to “naïve” cells analyzed from cell culture or from tumors. Actually, “evolved” populations show a basal level of NAD that is ~2‐fold lower than in “naïve” cells (Figure 6a). This reduction in NAD was stable and persisted over several months of culture. In parallel, we monitored the activity of various components of the respiration in “naïve” and “evolved” cells using the Seahorse technology. We found that “evolved” cells have a significantly lower OXPHOS as well as glycolysis function compared to their respective “naïve” counterpart (Figure 6b).

## 4 DISCUSSION

Increasing the mutation rate by genotoxic therapies places surviving cells under intense evolutionary selective pressure, favoring Darwinian dynamics (Nesse, 2001). Indeed, resistant cells are initially present in the tumors to a lesser extent or are generated during treatment (by the drug's mutagenic effect) and then positively selected under the pressure of treatment. However, the environment conditions are different for development of intrinsic resistance acquired during tumor development and therefore being a side effect of oncogenesis, and acquired resistance mainly driven by the drug selective pressure. It has been shown that tumor cells that are resistant to AsiDNA show few modifications in repair pathways and cell cycle and are more similar to nontumor cells than the cell lines sensitive to AsiDNA (Jdey, Thierry, Russo, et al., 2017). This suggests that to acquire resistance during treatment, sensitive cells will have to reverse to some kind of normality that seems difficult to achieve. To further analyze the characteristic of the acquired resistance to AsiDNA, cells were exposed to long‐term cyclic treatment which was successful in selecting resistant clones with different treatments but not with AsiDNA (Jdey et al., 2019). Not only these protocols failed to promote resistance to AsiDNA but they induce a unique evolution of the treated population that lead cells to a state in which they acquire a higher sensitivity to the selecting drug. This evolution toward sensitivity was observed in several cell lines and seems to be a common feature of cells growing with AsiDNA.

In this manuscript, we characterize the modifications of the population “evolved” under AsiDNA treatment. Genetic analysis of the population does not reveal a modification of the population that could suggest a clonal expansion. Moreover, the chromatin modification and large transcriptional change observed in independent “evolved” populations suggest a common epigenetic change associated with energy metabolism down‐regulation and NAD depletion that could be responsible for the acquired sensitivity to AsiDNA. Surprisingly, the new state of the population does not confer any advantage in proliferation and cell cycle and increases sensitivity to AsiDNA. Therefore, we would like to develop a theory that will have to be consolidated further. The high activity of PARP induced by its binding to AsiDNA transitory depletes cells from the NAD metabolite that is overused by the enzyme to synthetize polymers of ADP‐ribose (PAR). It results in a massive energy consumption which could reduce the ability of resistant cells to get advantage in the population taking in accordance with the evolution principle that any adaptation to improve fitness to an environment has a cost.

The process of carcinogenesis requires genetic instability and highly selective local microenvironments, the combination of which promotes somatic evolution. Under the selective pressure of chemotherapy, resistant populations of cancer cells invariably evolve, giving rise to “resistant clones” that have adapted to the new environment induced by the treatment. Here, we observed a general behavior of total population of tumor cells that appears to decrease their response to AsiDNA by decreasing the sensitivity of its target enzymes PARP and DNA‐PK. Such behavior is reminiscent of the ecological and evolutionary “smoke detector principle” proposed by Nesse (Nesse, 2001, 2005). It stipulates that although natural defenses (*e.g*., flight, cough, stress, anxiety, vigilance) should theoretically be expressed to a degree that is near the optimum needed to protect against a given threat, many are expressed too readily or too intensely. This is because when the cost of expressing an all‐or‐none defense is low compared to the potential harm it protects against, the optimal system will express many exaggerated responses. However, when the rate of false alarms becomes excessive, that is, the multiple modifications induced by the activation of PARP and DNA‐PK by AsiDNA, selection in return favors a reduction in the sensitivity of the response (Beauchamp & Ruxton, 2007; Nesse, 2005). For example, vigilant behavior in gregarious animals is costly in time and energy and it may be advantageous for individuals not to respond to all alarm calls when false ones become too frequent (Gillies, Verduzco, & Gatenby, 2012). Actually, a recent modeling approach of the signal detection theory demonstrates that responses to risk should depend on the state‐dependent background of mortality and could lead to decrease in responsiveness to alarm signal (Gillies et al., 2012). Because malignant cells are living entities, we propose that they should be driven by the same evolutionary logic.

However, though many repair genes are down‐regulated in “evolved populations” we did not identify a clear defect in damage signaling or DNA damage repair. This observation leads us to consider that the strongest danger for the tumor cells is the overconsumption of energy. Therefore, by down‐regulating many genes, the population would decrease its requirement in energy and protect the cells from moderate variations of resources, at the cost of a lower resistance to AsiDNA that induces high energy consumption.

Most of the results used in this study were obtained on the breast cancer MDA‐MB‐231 cell line. However, several of them were reproduced in other cell lines. In a general way, all the cell lines seem to evolve in the same way though in a more moderate extent than MD‐MB‐231 cell line. The fact that this cell line shows a high NAD basal level as compared to other cell lines suggests that some preexisting deregulations in metabolism could have increase the impact of its role in the evolution under AsiDNA treatment.

This work supports current evolutionary ideas according to which by leveraging our knowledge of cancer's evolution and its ecological nature we can come up with new strategies for treating it, by shaping its evolutionary path and potentially establishing long‐term tumor control (Silva et al., 2012; Ujvari et al., 2017).

## CONFLICT OF INTEREST

The authors have submitted patents for the AsiDNA technology.

## Supporting information

Table S2

Supplementary Material

## Data Availability

Transcriptome raw data are deposited in GEO (identifier GSE144023).
