# Supplementary material for: Evolution of tumor cells during AsiDNA treatment results in energy exhaustion, decrease in responsiveness to signal, and higher sensitivity to the drug
Source: Evol Appl. 2020 Apr 11;13(7):1673–80. doi: 10.1111/eva.12949 (PMC7428804; doi:10.1111/eva.12949)
Supplement: Supplementary file 2 — Supplementary Material [file EVA-13-1673-s002.doc]

**SUPPLEMENTARY MATERIAL**

**Supplementary Materials and Methods**

***Genomic analysis using SNP array***

Genomic analysis was conducted on Affymetrix Cytoscan HD arrays as described before (31). Briefly, 250ng of gDNA was used to perform Affymetrix Human Cytoscan HD assay following the manufacturer’s protocol. gDNA was digested with NspI enzyme. Dedicated adaptors were ligated to DNA before running 4 independent PCR reactions, using Titanium^TM^ Taq DNA polymerase (Clonetech). The PCR products were then controlled on 2% agarose gel, pooled, and fragmented using DNase I enzyme. Size of resulting DNA was checked by gel electrophoresis before terminal labeling and microarray hybridizations on Cytoscan HD array. After hybridization, microarrays were stained, washed and scanned using Affymetrix GCS3000 system. Data were controlled with Affymetrix Genotyping console: waviness SD were higher than 0.12 (mean = 0.18), SNPQC higher than 15.00 (mean 16.38) and MAPD were lower than 0.25 (mean = 0.15). Raw data were analysed using ChAS, a software offering intuitive and flexible workflow for accurate analysis and tailored for cytogenetics was developed with input from leading experts and is customized for copy number and cytogenetics research analysis and reporting. ChAS enables you to view and summarize chromosomal aberrations, including copy number gain or loss, mosaicism, or loss/absence of heterozygosity (LOH/AOH) across the genome.

***Expression array data analysis***

Affymetrix Human Gene 2.1 Array datasets were controlled using Expression console (Affymetrix) and further analyses and visualization were made using EASANA® (GenoSplice, [www.genosplice.com](http://www.genosplice.com/)), which is based on the GenoSplice’s FAST DB® release 2016_1 annotations (32)(33). Gene Array data were normalized using quantile normalization. Background corrections were made with antigenomic probes and probes were selected as described previously (34). Only probes targeting exons annotated from FAST DB® transcripts were selected to focus on well-annotated genes whose mRNA sequences are in public databases (32)(33). Bad-quality selected probes (e.g., probes labeled by Affymetrix as ‘cross-hybridizing’) and probes whose intensity signal was too low compared to antigenomic background probes with the same GC content were removed from the analysis. Only probes with a DABG P-value ≤ 0.05 in at least half of the arrays were considered for statistical analysis (34). Only genes expressed in at least one compared condition were analyzed. To be considered to be expressed, the DABG P-value had to be ≤ 0.05 for at least half of the gene probes. We performed an unpaired Student’s t-test to compare gene intensities in the different biological replicates. Genes were considered significantly regulated when fold-change was ≥ 2 and uncorrected P-value.

**Table S1**

**Supplementary TABLE**

naïve evolved


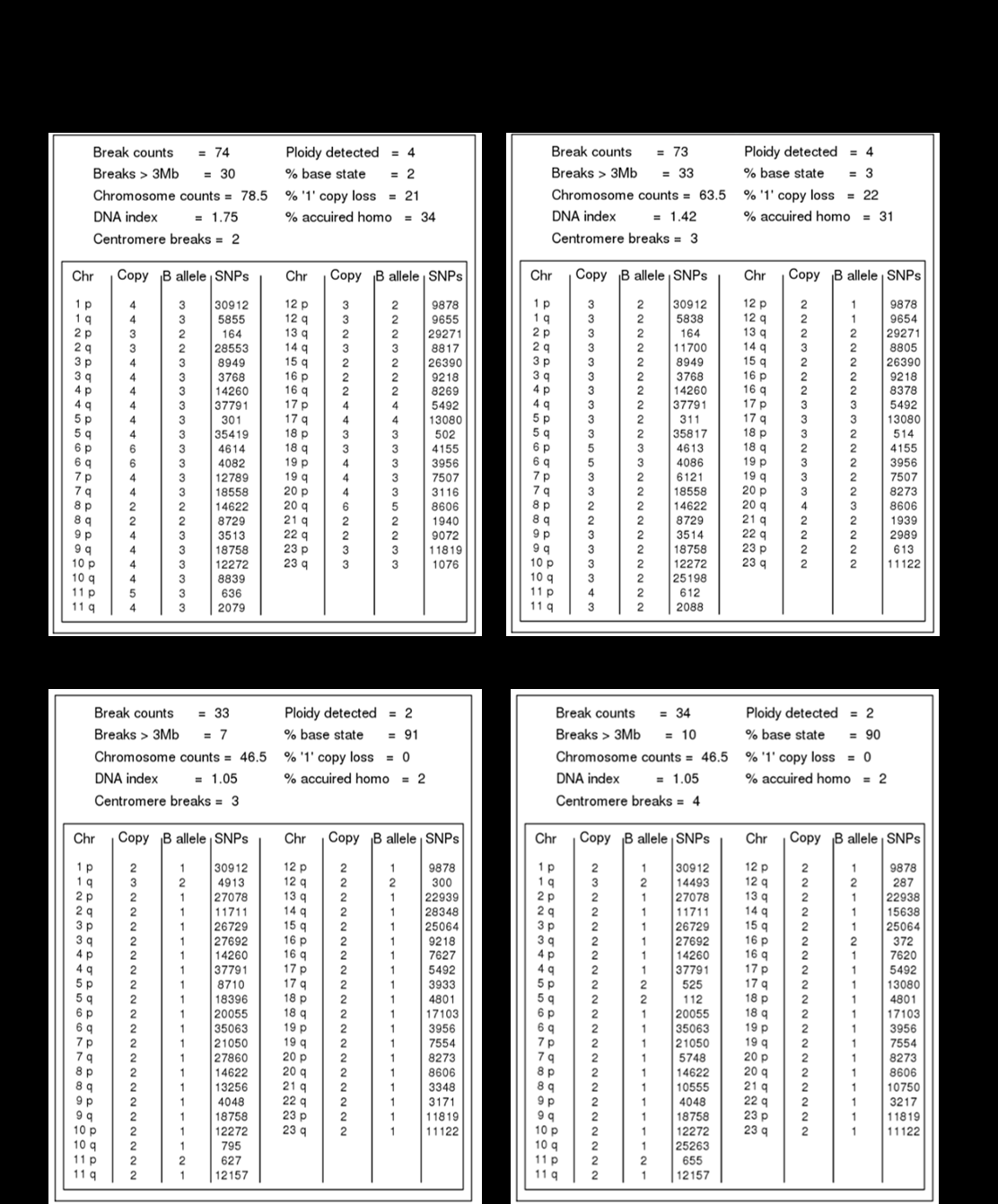


**Supplementary Table S1. Effect of AsiDNA repeated treatments on cell genomic status.** Genomic DNA extracted from “naïve” and “evolved” populations (3 independent cultures for each condition) was analysed using Affymetrix Cytoscan HD arrays as described in supplementary Material and Methods. Genomic profiles of (A) tumor (MDA-MB-231) and (B) non-tumor (MCF-10A) “naïve” cells (^NT^) or “evolved” (AsiDNA^3C^).
